# Combined impacts of warming and methomyl on neurophysiological and behavioral responses in Amazonian frog tadpoles

**DOI:** 10.1007/s10646-025-03022-3

**Published:** 2026-02-18

**Authors:** Guilherme Azambuja, Samara Silva de Souza, Carina Brunehilde Silva, Igor Luis Kaefer, Adalberto Luis Val, Daiani Kochhann

**Affiliations:** 1 Postgraduate Program in Zoology, Department of Biology, Federal University of Amazonas, Manaus, Brazil; 2 Laboratory of Ecophysiology and Molecular Evolution, Brazilian National Institute for Research of the Amazon BR, Manaus, Amazonas Brazil; 3 Center of Exact and Earth Science, Acarau Valley University, Sobral, Ceará Brazil; 4 Institute of Mathematics, Statistics and Scientific Computing, University of Campinas, Campinas, São Paulo, Brazil; 5 Laboratory of Behavioural Ecophysiology, Center of Agrarian and Biological Sciences, Acaraú Valley University, Sobral, Ceará Brazil

**Keywords:** Anuran larvae, Climate change, Ecotoxicology, Pollution

## Abstract

Amphibian populations are declining globally due to anthropogenic stressors such as climate change and environmental contamination. In Amazonia, where aquatic species already endure conditions near their thermal thresholds, the combined effects of rising temperatures and pesticide exposure pose a threat to their survival and physiological stability. Here, we evaluated the isolated and interactive impacts of elevated temperature and sublethal concentrations of the carbamate pesticide methomyl on behavior, neurophysiology, and endocrine responses in tadpoles of two Amazonian frogs, *Osteocephalus taurinus* and *Scinax ruber*. Tadpoles were exposed to the stressors for 96 h, and the subsequent assays measured substrate preference, locomotor and antipredator behavior, acetylcholinesterase (AChE) activity, and whole-body cortisol concentrations. *O. taurinus* showed significant behavioral impairments under both stressors, including reduced swimming activity and increased immobility on dark substrates, suggesting antipredator strategies. AChE activity decreased under elevated temperature but showed a pronounced increase under combined exposure to methomyl and heat, while cortisol concentration did not change significantly. In contrast, *S. ruber* maintained stable behavior and enzyme activity, yet exhibited robust endocrine responses, with significant cortisol elevation under both stressors. No significant responses to alarm substances were observed in either species. Our results highlight distinct species-specific responses: *O. taurinus* displayed greater behavioral sensitivity, whereas *S. ruber* showed pronounced endocrine reactivity. These divergent strategies likely reflect ecological and physiological adaptations, emphasizing the importance of multi-stressor and multi-species approaches in risk assessment. This study underscores the vulnerability of tropical amphibians to co-occurring climate and pollution stressors and calls for integrative conservation strategies that consider species-specific traits and ecological histories.

## Introduction

Amphibians face unprecedented threats to their survival, with substantial population declines linked to the intensification of human activities (Green et al. 2020; Nolan et al. 2023). The escalating exploitation of natural resources, driven by technological advances and human population growth, has increased anthropogenic pressure on aquatic ecosystems, which are critical habitats for many amphibian species. Amphibians are among the most threatened vertebrates, and their declines are increasingly attributed to climate change and environmental pollution (Balvanera et al. 2020; McRae et al. 2020). Their sensitivity to environmental disturbances makes them valuable bioindicators, owing to their intrinsic morphophysiological traits: (I) eggs that require moist environments, (II) an aquatic larval stage in most species, and (III) highly permeable skin, which facilitates the absorption of contaminants (Slaby et al. 2019). Furthermore, anuran metamorphosis represents a critical period during which specialized larval structures (e.g., gills and tail) are resorbed, while terrestrial organs (e.g., lungs, limbs) develop (Gosner 1960; Yaoita 2019), rendering them particularly vulnerable to environmental disruptions.

In recent decades, pesticide contamination of aquatic ecosystems has exposed non-target species to toxic compounds. Global pesticide usage surged from 2.18 million tons in 2000 to 3.69 million tons in 2022, a 69.68% increase in just 22 years (FAO 2025). These contaminants enter aquatic environments through spray drift during aerial application, direct overspray, surface runoff from heavy rainfall, and soil erosion (Solomon and Thompson 2003; Commelin et al. 2022). In Brazil, one of the most widely sold pesticides is the carbamate insecticide methomyl, with 9.1 million tons commercialized in 2023, a 23.6% increase over the past decade (IBAMA, 2025). Methomyl is extensively used to control a broad spectrum of insect pests. Despite being banned in several countries due to its high toxicity to vertebrates, its use is permitted in Brazil (Lin et al. 2020; NCBI, 2024). Its physicochemical properties, including high water solubility (57.9 g/L⁻¹), low sediment adsorption, and extended half-life (6 days in surface water, 25 weeks in groundwater), enhance its environmental mobility, persistence and bioavailability to aquatic organisms (Selvam et al. 2013; Zheng et al. 2023; Dantas et al. 2024). Methomyl exerts its toxic effects by inhibiting acetylcholinesterase (AChE), leading to the accumulation of acetylcholine (ACh) in synaptic clefts, which, in vertebrates, triggers neurotoxic effects such as muscle tremors, spasms, and paralysis (Van Scoy et al. 2013; NCBI, 2024). In non-target organisms, methomyl is associated with neurotoxicity, endocrine disruption and behavioral impairments. In fish, exposure reduces AChE activity, elevates cortisol concentrations and compromises locomotor performance (Jablonski et al. 2022; Rahman et al. 2022; Li et al. 2024). In tadpoles, pesticide-induced behavioral alterations may impair energy acquisition, a critical factor for proper growth and metamorphosis. Additionally, locomotor deficits could heighten predation risk, thereby reducing survival (Freitas et al. 2017; Liu et al. 2021). Although previous studies have documented neurotoxicity, cellular damage and behavioral impairments caused by methomyl in tadpoles of species such as *Bufo arabicus* (Seleem 2019a,b) and *Hoplobatrachus rugulosus* (Trachantong et al. 2017), the impacts of this pesticide on Neotropical tadpoles remain largely unexplored. This knowledge gap is critical because amphibians in Amazonia play pivotal ecological roles as regulators of trophic dynamics, contributors to nutrient cycling and as indicators of ecosystem health (Valencia-Aguilar et al. 2013; Azambuja et al. 2024). Moreover, the Neotropics harbor unparalleled amphibian diversity, encompassing a wide range of ecological strategies and physiological adaptations that can modulate how different species respond to environmental stressors (Guerra et al. 2020; Azambuja et al. 2024). Comparative approaches are therefore essential to elucidate how distinct lineages cope with multiple stress factors. Previous studies have demonstrated asymmetric responses, where co-occurring species exhibit contrasting behavioral or physiological outcomes under the same contaminant exposure, reflecting differences in life-history traits and evolutionary background (Boone and Semlitsch 2002; Distel and Boone 2011a,b; Costa et al. 2024; Henao et al. 2022).

The impact of climate change on tropical ecosystems such as Amazonia is of growing concern, especially for aquatic organisms which already experience temperatures near their thermal limits (Campos et al. 2021; IPCC, 2021). For amphibians, whose thermoregulation depends on environmental conditions, warming directly affects essential physiological processes and behavior (Watkins and Vraspir 2006; Navas et al. 2008). Elevated temperatures may exacerbate physiological dysfunctions, such as locomotion impairment due to increased energy demands (Ruthsatz et al. 2018; Gallo et al. 2020). Amphibian larvae are known to exhibit interactive effects when exposed to multiple environmental stressors, where factors such as predation risk, density, or physicochemical alterations can intensify contaminant toxicity (Boone and Semlitsch 2001; Relyea and Mills 2001; Burraco and Gomez-Mestre 2016). Despite this, the synergistic effects of pesticides combined with temperature remain poorly studied. One of the few available pieces of evidence shows that rising temperatures enhanced methomyl toxicity in three Asian anuran species (Lau et al. 2015). However, as reviewed by Azambuja et al. (2024), no studies have addressed this interaction in Amazonian species. This is critical because many Amazonian ectotherms already live near their CT_max_, resulting in narrow thermal safety margins and increased vulnerability to climate stress (Simon et al. 2015; Campos et al. 2021). This knowledge gap is critical, given that the Amazonia region already faces pesticide contamination (Rico et al. 2022; Cezarette et al. 2024) and accelerated warming (IPCC, 2021; Braz-Mota and Luis Val 2024), and these factors may impact behavior, physiology and larval development (Baier et al. 2016; Quiroga et al. 2019, 2024). Therefore, this study evaluates the isolated and combined effects of methomyl and elevated temperature on behavioral, neurophysiological and endocrine parameters in the tadpoles of *Osteocephalus taurinus* (Steindachner, 1862) and *Scinax ruber* (Laurenti, 1768), two Amazonian hylid frog species with distinct ecological backgrounds and environmental tolerances. We predicted that (i) exposure to methomyl, elevated temperature or their combination would impair neurophysiological and endocrine responses, characterized by acetylcholinesterase (AChE) inhibition and cortisol elevation; (ii) such physiological disturbances would lead to behavioral alterations, including changes in substrate preference and reduced swimming performance; and (iii) the ability of tadpoles to detect and respond to alarm substances would be compromised under methomyl exposure, elevated temperature, and particularly their combination, reflecting cumulative neuroendocrine disruption.

## Materials and methods

### Clutch collection and maintenance

*Osteocephalus taurinus* and *Scinax ruber* are distributed throughout the Amazon basin in Brazil. These species reproduce year-round, with peak activity during the rainy season when they lay their eggs in temporary aquatic environments. *O. taurinus* deposits its eggs on the surface of ponds, where they are encased in a gelatinous film, while *S. ruber* deposits its eggs attached to vegetation or along the margins of ponds. *S. ruber* is commonly found in open areas, forest edges and clearings, and is frequently observed in trees and anthropogenic environments (Lima et al. 2012; Frost 2021). Tadpoles of *O. taurinus* and *S. ruber* were collected at Gosner stages 24–25 (Gosner 1960) from temporary ponds within the Adolpho Ducke Forest Reserve (2.916° S, 59.883° W), and in a forested area of the National Institute for Amazonian Research (INPA) campus (3.097° S, 59.987° W), Manaus, Amazonas, Brazil. Both locations are surrounded by vegetation cover and lack nearby agricultural activity, as confirmed by land-cover data from MapBiomas (2025), indicating no direct exposure to pesticide runoff or agricultural contaminants. For each species, tadpoles were collected and transported to the laboratory and kept in 70-L aquaria (70 × 50 × 30 cm) with a temperature of 26.13 ± 0.47 °C, oxygen 5.72 ± 1.26 mgO2 L-1, and pH 7.12 ± 0.38, at a density of three tadpoles per liter for at least 14 days for acclimation to lab conditions. A 12:12 light: dark cycle was used to maintain the photoperiod, with water renewals occurring every 48 h. Tadpoles from different clutches were randomized in the aquaria to ensure sample variability. They were fed daily with an *ad libitum* diet comprising a blend of commercial fish feed and rabbit feed, containing 32% and 14% protein, respectively.

### Determination of LC₅₀ values

The LC₅₀ determination was conducted in a separate preliminary study designed to support the present experiment. Acute toxicity tests were performed for both species (*O. taurinus* and *S. ruber*) at 26.5 °C and 30 °C, using the commercial formulation Lannate^®^ as the methomyl source. Ten tadpoles were placed in each aquarium (22 × 20 × 18 cm; 6 L), and each concentration was tested in triplicate. The exposure lasted 96 h, during which mortality was recorded every 24 h. LC₅₀ values were estimated for each temperature and species using Probit analysis, following OECD guidelines for acute toxicity tests (OECD, 2025). These LC₅₀ data served as the basis for selecting the sublethal exposure level (10% of the 96-h LC₅₀) used in the present study.

### Exposure to sublethal concentrations of methomyl at different temperatures

Ten tadpoles at Gosner stages 25–28 (Gosner 1960) were randomly selected and placed into experimental aquaria (22 × 20 × 18 cm; 6 L), with each concentration tested in duplicate, totaling 20 tadpoles per concentration. Following their transfer, tadpoles were given a 30-minute acclimation period before initiating the heating process. Water temperature was gradually increased from the acclimation baseline (26 °C) to the target values for each aquarium (26.5–30 °C) at a rate of 0.03 °C min⁻¹, following established protocols (Fan et al. 2021; Azambuja et al. 2021; Kochhann et al. 2021) and representing ecologically realistic warming rates observed in shallow ponds (Terblanche et al. 2011; Turriago et al. 2022). The temperature regimes represented: (1) 26.5 °C, mimicking natural temporary pond conditions, and (2) 30 °C, corresponding to the IPCC SSP3–7.0 projected warming scenario (+ 3.5 °C global average; IPCC, 2021).

After each aquarium reached its target temperature, the calculated volume of the commercial methomyl formulation (Lannate^®^) required to achieve the nominal concentrations (mg L⁻¹) was added directly to the water containing the tadpoles. The solution was homogenized by continuous aeration to ensure uniform distribution of the compound. Methomyl is characterized by high solubility, low volatility, and chemical stability under the experimental conditions, with a reported half-life of approximately 25–30 days in freshwater at neutral pH and in the absence of direct UV light (Van Scoy et al. 2013; Dantas et al. 2024). These properties ensured stable exposure concentrations throughout the 96-hour experimental period.

Tadpoles were exposed to 0 mg a.i./L (control) and 10% of the LC₅₀. The sublethal concentration employed corresponded to 10% of the previously determined 96-h LC₅₀ for each species and temperature. This fraction is widely used in ecotoxicological studies to assess sublethal effects without inducing mortality (Al-Asgah et al. 2015; Jiménez et al. 2021; Liu et al. 2021; Uçkun and Özmen 2021). For *O. taurinus*, the sublethal concentrations were 9.64 mg/L (26.5 °C) and 4.59 mg/L (30 °C). For *S. ruber*, the sublethal concentrations were 1.55 mg/L (26.5 °C) and 1.97 mg/L (30 °C).

Throughout the 96-h exposure, water parameters remained stable. For *O. taurinus*, mean temperature was 26.5 ± 0.12 °C and 30.0 ± 0.20 °C, dissolved oxygen averaged 88.8 ± 2.3% and 88.3 ± 2.6%, and pH was 7.30 ± 0.09 and 7.19 ± 0.07 at 26.5 °C and 30 °C, respectively. For *S. ruber*, mean temperature was 26.5 ± 0.12 °C and 30.1 ± 0.15 °C, dissolved oxygen 88.4 ± 0.8% and 89.0 ± 1.9%, and pH 7.35 ± 0.24 and 7.52 ± 0.24 at 26.5 °C and 30 °C, respectively. All individuals were unfed during the 96-h exposure, ensuring a standardized fasting state.

### Preparation of alarm substance

The alarm substance was prepared from a single naïve tadpole (~ 85 mg wet mass; not previously exposed to methomyl or temperature variation) of the same species as the tested individuals, ensuring intraspecific specificity. The donor tadpole was euthanized by double pithing of the spinal cord, and the body was manually macerated with a scalpel in 100 mL of distilled water, yielding a final extract concentration of approximately 0.85 mg mL⁻¹. The solution was used immediately after preparation, following established procedures for alarm cue preparation in anuran larvae (Elvidge et al. 2023; Lipkowski et al. 2024).

### Behavioral assessment of tadpoles

Following the 96-hour acute exposure to methomyl and/or temperature, behavioral assays were conducted under controlled environmental conditions. Tests were performed in an isolated and quiet room used exclusively for behavioral trials, minimizing external visual and acoustic disturbances. All assays were carried out during daytime, maintaining the natural photoperiod of the region (12 h light / 12 h dark), and at the same temperatures, pH, and dissolved oxygen levels as the exposure phase to ensure environmental consistency. To standardize exposure duration across individuals, a subset of eight tadpoles per treatment group was randomly selected for testing.

The substrate preference test was conducted in circular arenas (20,5 cm) with 2 cm water depth (420 ml) containing a bipartite black-and-white substrate (50% each), following the protocol adapted from Guimarães et al. (2021). After introducing individual tadpoles into the arena, a 10-minute acclimation period was provided prior to 20 min of video recording. Video analysis quantified: (1) time spent in each substrate zone and (2) duration of swimming activity.

The alarm response assay was conducted in rectangular tanks (15 × 22.2 cm) with 2 cm water depth (475 mL). Individual tadpoles were acclimated for 10 min before sequential additions of (1) 10 mL of distilled water (vehicle control; ≈2.1% of total volume) and, after 10 min, (2) 10 mL of the conspecific alarm substance prepared at a ratio of one tadpole per 100 mL immediately before use. Additions were performed slowly using a 10 mL syringe, releasing the liquid along the tank wall over approximately 45 s to minimize hydrodynamic disturbance. Behavioral responses were recorded for 30 min in total (10 min baseline, 10 min post-vehicle, and 10 min post-alarm). Video analyses were conducted in BORIS software (Friard and Gamba 2016) to quantify (1) spatial use patterns (center vs. corner occupancy) as stress indicators and (2) changes in swimming activity post-exposure. Tadpoles were unfed during the 96-h exposure period preceding the behavioral tests, ensuring a standardized fasting state across treatments. Each tadpole was subjected to only one behavioral assay to avoid carryover effects between tests. At the end of each behavioral assay, the tadpoles were anesthetized with MS-222 and euthanized. All the individuals were preserved at -80 °C. Tadpoles from the substrate preference test were later used for acetylcholinesterase (AChE) analysis, while those from the alarm response assay were used for cortisol quantification.

### Biochemical responses

The activity of whole-body acetylcholinesterase (AChE) was measured using the method described by Ellman et al. (1961). Body samples were homogenized (1:4 w/v) in a 0.1 M phosphate buffer with 20% glycerol, pH 7.4, and then centrifuged for 15 min at 850 xg at 4 °C. The supernatant was collected and centrifuged again at 12.800 xg at 4 °C. Acetylcholine iodide (9 mM) was used as the substrate, and DTNB served as the color reagent. AChE enzyme activity was measured using a spectrophotometer (SpectraMax M2, Molecular Devices) at a wavelength of 412 nm and was expressed as nmol min^− 1^ mg protein^− 1^.

The total protein content in the whole-body samples was quantified in a spectrophotometer (SpectraMax M2, Molecular Devices) at 595 nm using bovine serum albumin (BSA) as the standard and in accordance with Bradford (1976).

The concentrations of whole-body cortisol were measured using a commercial cortisol ELISA Kit (Cayman Chemical, Item No. 500360). To obtain a homogenized sample, the tadpole was crushed using an ultrasonic homogenizer (Branson Sonifier 450, VWR Scientific). Between tests, the mixer was rinsed with ethanol to avoid cross-contamination. Subsequently, 5 mL of methanol was added as the extraction solvent. The sample was mixed in a vortex (MAXIMIX Plus, Thermolyne) for 30 s, placed on an overhead shaker (TE 420, TECNAL) at 180 rpm for 1 h at room temperature, and centrifuged for 10 min at 3500 xg at 7 °C (Centrifuge 5430 R, Eppendorf). The entire supernatant was collected, evaporated to a concentrate under vacuum (Concentrator Plus, Eppendorf), and reconstituted in 200 µL ELISA Buffer (1X) from the commercial kit. The assay was developed following the protocol provided by Cayman Chemical (Cortisol ELISA Kit, Cayman Chemical Item No. 500360). Samples were read and quantified in duplicate using a microplate reader (SpectraMax M2, Molecular Devices) at an absorbance of 405 nm.

### Statistical analyses

All the statistical analyses were performed using R software (version 4.2.2). We fitted linear mixed models including an aquarium-level random effect using the lme4 package in all analyses. This hierarchical approach treats the aquarium as the experimental sampling unit and the tadpoles within each aquarium as nested observations, thus accounting for the dependence structure inherent to the design and partitioning variance within and between aquaria. This approach provides insights not only into the variance components but also into the direction of the fixed effects.

However, these results can be unstable due to n and the violation of the normality assumption in some variables of interest. To address these issues, we adopted a two-step inference approach, combining parametric mixed-model results with a nonparametric permutation test using the *permuto* package. This approach provides robust inference under potential violations of normality, small-sample conditions, and unbalanced designs. The permutation test outcomes guided final interpretations.

For activity/inactivity time data, our goal was to examine main effects and interactions between methomyl presence/absence, temperature, substrate preference, and their interactions. Alarm substance responses were evaluated to compare: (1) changes in swimming activity (time moving) and (2) shifts in spatial distribution (center vs. corner occupancy) between pre- and post-stimulus periods. In the latter case, a random effect was also assigned to the individual tadpole to account for repeated measurements within subjects. Acetylcholinesterase (AChE) activity and cortisol concentration were also analyzed in relation to methomyl exposure and temperature. All analyses described below were performed separately for *O. taurinus* and *S. ruber*.

## Results

### Survival after sublethal exposure

No mortality was recorded in any treatment throughout the 96-h exposure period.

### Substrate preference

In this comparative study, we decided to analyze activity and resting time separately, since they perform differently, and summing the total time masks the covariate effects.

All fitted linear mixed models revealed a singular fit, with the variance of the random term (aquarium) estimated as zero, indicating that between-aquarium variance could not be reliably estimated, likely due to the limited number of replicates per treatment, for both activity and inactivity times in the two species, except for *S. ruber* in activity time, where the variance of the random effect was 153.3 (sd = 12.38). This result suggests the absence of significant variability between aquaria within the same treatment.

### Swimming activity

**Fig. 1:**
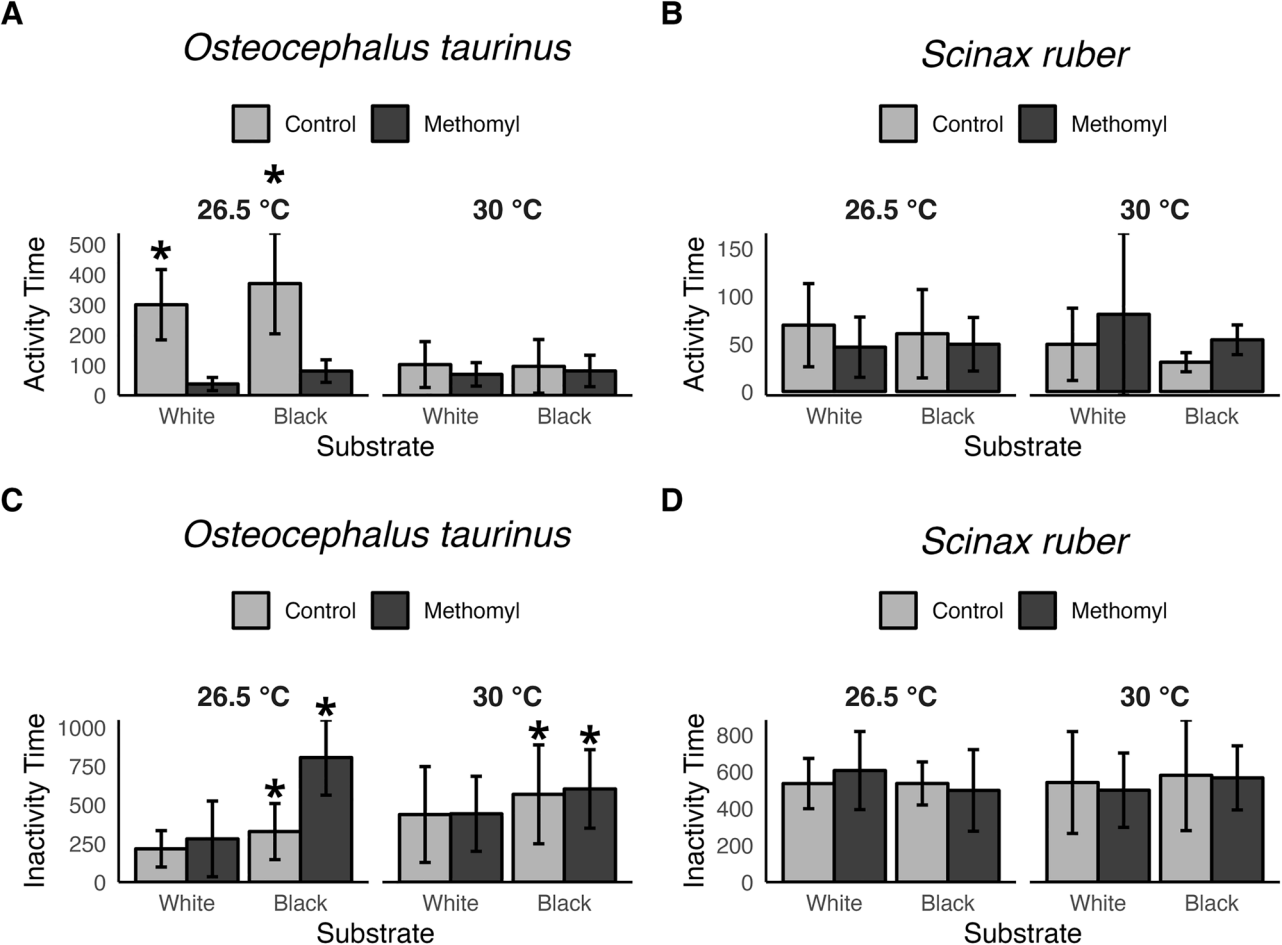
Activity time (**A**–**B**) and inactivity time (**C**–**D**) of *Osteocephalus taurinus* and *Scinax ruber* tadpoles (in seconds), respectively, on white and black substrates under different temperature and methomyl exposure conditions. Bars represent raw group means ± SD. * indicate statistically significant differences (p < 0.05)

Methomyl exposure and elevated temperature independently reduced swimming activity in *O. taurinus* tadpoles (methomyl: *p* < 0.001; temperature: *p* < 0.001). A significant interaction revealed stronger methomyl effects at 26 °C (*p* = 0.002) (Fig. 1A). In *S. ruber* tadpoles, activity levels were unaffected by methomyl exposure (*p* = 0.43), temperature (*p* = 0.49), or interaction between methomyl and temperature (*p* = 0.09) (Fig. 1B). However, neither species shows a significant preference for either substrate (*O. taurinus*: *p* = 0.26; *S. ruber*: *p* = 0.34).

### Resting behavior

*Osteocephalus taurinus* tadpoles exhibited substrate-dependent inactivity, spending more time resting on black substrates (*p* = 0.002) (Fig. 1C). In contrast, no other covariate significantly affected inactivity duration for this species. Similarly, no significant effects were detected in *S. ruber* tadpoles (Fig. 1D).

### Alarm substance response

In this context, all fitted linear mixed models estimated the variance of the random term (aquarium) as zero, except for *S. ruber* in swimming activity, where the variance of the random effect was 3373 (sd = 58.08). This suggests that between-aquarium variance could not be reliably estimated, likely due to the limited number of replicates per treatment, and does not confirm the absence of variability among aquaria within the same treatment.

Following alarm substance exposure, neither swimming activity nor spatial distribution (center vs. edge preference) showed significant changes for *O. taurinus*. Conversely, swimming activity in S. ruber tadpoles was significantly affected by methomyl presence (*p* = 0.02), reflected by increased activity time, while spatial distribution and its interaction with activity remained non-significant.

### Biochemical responses

#### Acetylcholinesterase activity

The random-effect variance was null in all models, and one tadpole observation was missing in the high-temperature, no-methomyl treatment due to technical issues, leaving only seven individuals in that aquarium. Nevertheless, because all analyses used permutation-based inference, the potential bias due to this minor imbalance was mitigated. Additionally, the null random-effect variance reflects an estimation limitation, rather than the confirmed absence of variation among aquaria.

**Fig. 2:**
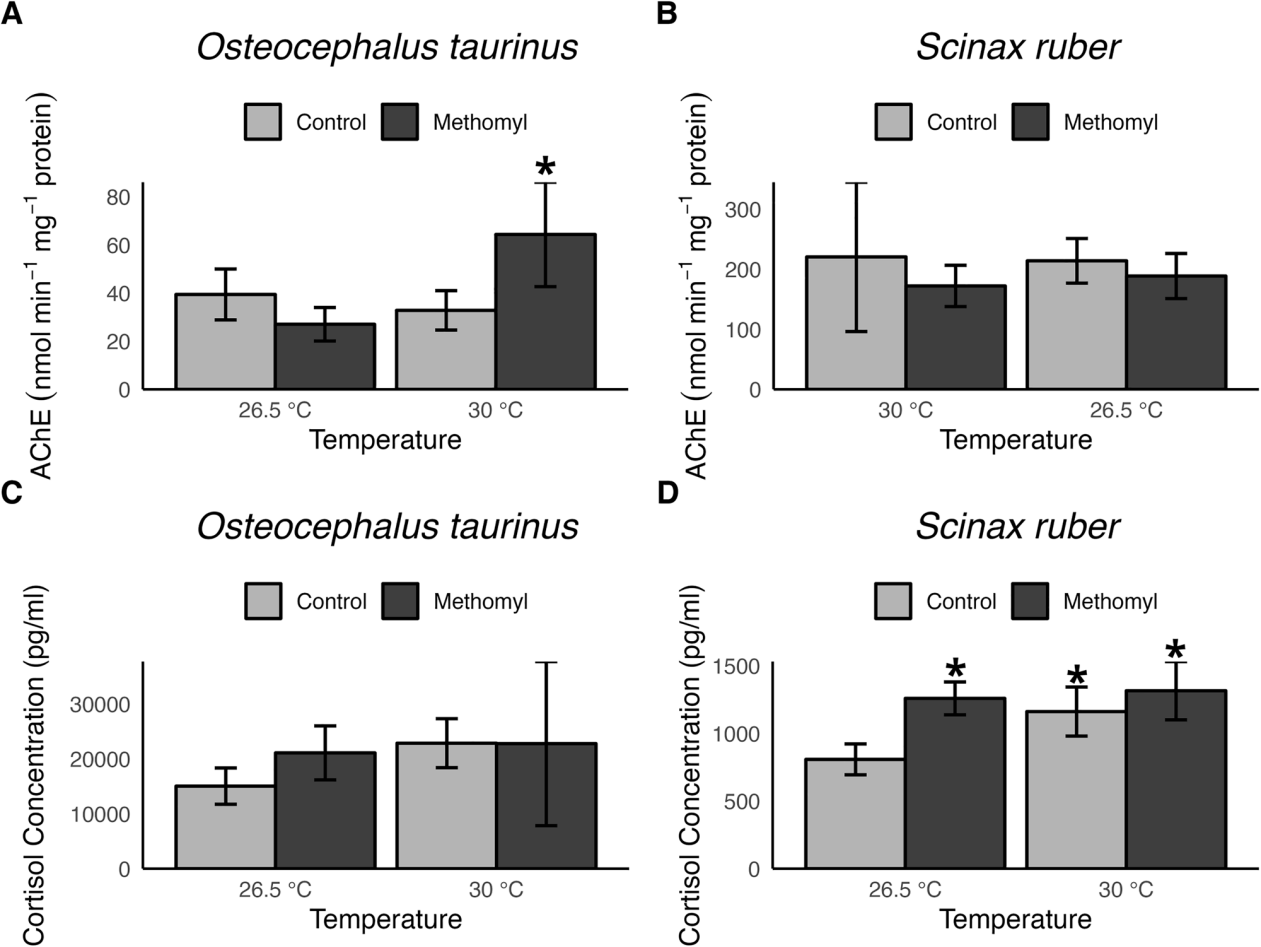
Physiological responses of *Osteocephalus taurinus* (**A**, **C**) and *Scinax ruber* (**B**, **D**) tadpoles to temperature elevation and methomyl exposure on AChE activity and cortisol concentration. Bars represent raw group means ± SEM. * indicate statistically significant differences (p < 0.05)

Regarding AChE modulation in *Osteocephalus taurinus*, a significant interaction between temperature and methomyl exposure (*p* < 0.001) indicated increased activity at 30 °C under methomyl presence, while the baseline group (without methomyl) showed similar activity across temperatures. Separately, only temperature had a significant effect (*p* = 0.009). *Scinax ruber* maintained stable AChE activity across all the treatments (Fig. 2B).

### Cortisol response to stressors

In *O. taurinus*, there are no detectable effects from any of the stressors (Fig. 2C). In contrast, *S. ruber* showed significant increases in cortisol levels in response to all stressors: high temperature and methomyl exposure significantly increased cortisol levels (*p* < 0.001 and *p* = 0.006, respectively), and their interaction showed a non-additive effect (*p* = 0.04) (Fig. 2D).

## Discussion

Our results suggest significant interspecific differences in behavioral and physiological responses between *Osteocephalus taurinus* and *Scinax ruber* tadpoles to methomyl exposure and elevated temperature.

These disparities reflect species-specific adaptations to distinct environmental pressures. Among the multiple factors that influence microhabitat selection in lentic environments, substrate coloration may serve as a defense mechanism against visually oriented predators. Preference for low-contrast substrates enhances survival by reducing body visibility, particularly under high predation pressure from the aquatic insects commonly found in these habitats (Kang et al. 2015; Jara 2016). *O. taurinus* tadpoles spent significantly more time resting on dark substrates, indicating a substrate-dependent antipredator strategy. This behavioral pattern suggests a form of crypsis through immobility, enhancing camouflage effectiveness against visually oriented predators. Such microhabitat use minimizes exposure risk in high-predation environments, as dark substrates reduce body contrast in dark-pigmented tadpoles (Espanha et al. 2016; Eterovick et al. 2018; Eterovick et al. 2020). Substrate selection in tadpoles is not solely color-driven but also modulated by perceived predation risk. In temporary ponds, where predator density is high and structural refuges are scarce, larvae often rely on visual concealment strategies such as substrate matching and immobility (Touchon and Warkentin 2008). Experimental evidence supports this pattern: tadpoles exposed to predator chemical or visual cues preferentially occupy shaded or less exposed areas (Hettyey et al. 2011; Hettyey et al. 2015). Field observations during our collections corroborate this interpretation, as the studied ponds harbored numerous visually hunting predators (e.g., belostomatids, odonate larvae). A similar selection of low-contrast substrates has been reported for *Dryophytes plicatus* (Sánchez-Sánchez et al. 2023) and *Physalaemus gracilis* (Ximenez et al. 2012). Dark substrates may also confer thermoregulatory benefits, optimizing thermal balance for larval development. In contrast, *S. ruber* exhibited no clear substrate preference, possibly reflecting a background-matching strategy based on color plasticity, similar to that observed in *Pelobates cultripes*, which adjusts its pigmentation to track environmental brightness (Liedtke et al. 2023). Although further research is needed to confirm this mechanism in *S. ruber*, our preliminary field observations suggest that this species may undergo skin pigmentation changes, allowing it to explore a broader range of microhabitats. This plasticity may also be linked to thermoregulatory mechanisms, as observed in *Alytes dickhilleni* tadpoles, which modulate skin pigmentation based on ambient temperature (Rodríguez-Rodríguez et al. 2020). The results suggest that camouflage is not universal among Amazonian tadpoles. For instance, *Dendropsophus minutus* preferred substrates with intermediate contrast, while species such as *Callimedusa tomopterna*, *Leptodactylus fuscus*, *L. knudseni*, and *Rhinella marina* selected lighter substrates that increased contrast with their bodies (Guimarães et al. 2021). These findings highlight the diversity of antipredator strategies among species.

*Osteocephalus taurinus* exhibited reduced swimming activity under both methomyl exposure and elevated temperature. A significant interaction revealed stronger methomyl effects at 26 °C. In ectotherms, acute stress-induced lethargy is often interpreted as an adaptive trade-off, where individuals redirect finite energy resources away from locomotion and toward essential maintenance and detoxification processes (Sokolova 2013). The elevated temperature alone increases basal metabolic rate, demanding more energy for basic homeostasis (Gangloff and Telemeco 2018). When combined with a pesticide, this energetic demand is exacerbated by the costs of biotransformation, excretion, and cellular repair (Sokolova et al. 2012). Thus, the observed reduction in swimming may be a strategic down-regulation of activity to conserve energy in a scenario of heightened demand. However, this energy-saving strategy carries inherent ecological risks. Locomotor performance is critical for foraging and predator avoidance, directly impacting survival, growth and development (Bridges 1997). Swimming activity is closely linked to energy acquisition, as more active tadpoles have a higher likelihood of locating food, thus supporting adequate growth rates (Horat and Semlitsch 1994; Bridges 1997). Several studies have reported reduced locomotor activity in tadpoles exposed to carbamate and organophosphate insecticides, both known to inhibit AChE activity (Bridges 1997; Junges et al. 2017). Methomyl, absorbed through skin, gills, and gastrointestinal tract, reversibly inhibits AChE via carbamylation of the enzyme (Quaranta et al. 2009; David et al. 2017). This mechanism prevents acetylcholine (ACh) breakdown, prolonging its action in cholinergic synapses and triggering neurotoxic and behavioral effects, such as movement inhibition (Sparling and Fellers 2007). Contrary to our expectations, methomyl exposure did not reduce AChE activity; instead, AChE activity significantly increased at 30 °C when methomyl was present, while tadpoles in the baseline group (without methomyl) maintained similar activity across temperatures. Higher temperatures can enhance enzymatic activity by facilitating enzyme-substrate interactions, accelerating biochemical reactions, in accordance with thermodynamic principles (Lushchak 2011). This is especially relevant for tadpoles, whose metabolic processes are directly influenced by environmental temperature (Gangloff and Telemeco 2018). In this context, elevated temperatures raise metabolic rates, increasing the demand for AChE in order to prevent neuronal overstimulation due to excess ACh in synapses. AChE itself may recover more rapidly from methomyl inhibition under higher metabolic rates, possibly explaining the increased activity at 30 °C. Furthermore, although methomyl is an AChE inhibitor, its effects are reversible and less persistent than those of organophosphates (Fukuto 1990). Thus, higher temperature may accelerate the decarbamylation of AChE, restoring its function. This aligns with recent findings that temperature increases the kinetics of metabolic reactions, potentially accelerating the recovery of enzymatic functions in the presence of inhibitors, as temperature-driven acceleration of toxicokinetic processes has been observed in other organisms exposed to environmental stressors (Grenc et al. 2025). Increased metabolic rates, due to higher temperature, likely enhance the elimination and recovery rates of substances like methomyl, which modifies the enzyme’s ability to return to baseline activity more quickly than expected.

However, despite this enzymatic recovery, reduced swimming activity suggests that the response to stressors is not solely linked to AChE function. The lack of correlation between enzyme activity and behavior suggests that other mechanisms, such as increased energetic demands or hormonal imbalances, may also be involved and therefore warrants further investigation. This is consistent with the broader understanding that enzymatic function is not the only determinant of behavioral outcomes in ectothermic species exposed to multiple stressors. While the temperature may enhance enzymatic activity, the resulting behavioral effects can be more complex, influenced by factors such as energy allocation, neurological regulation, and neuroendocrine responses to thermal stress (DeLong et al. 2017). These findings suggest that the relationship between AChE activity and behavior may involve additional physiological responses that go beyond the enzymatic level. *S. ruber* showed no significant behavioral or enzymatic alterations under the same stressors, suggesting a lower susceptibility to methomyl, possibly due to inherently higher AChE activity. This pattern may reflect biochemical resistance rather than physiological resilience, although both mechanisms could contribute to the observed stability. This adaptive capacity may be related to its historical exposure to anthropogenic environments, where thermal fluctuations and contaminants are more frequent.

The absence of a response to the alarm substance observed across all treatments, including the control group, suggests that the evaluated species may not exhibit typical defensive behaviors triggered by this type of chemical signal, or that the stimulus used was not sufficiently effective or ecologically relevant to elicit a detectable reaction. The use of a single individual to prepare the alarm substance extract may have limited the signal intensity and ecological relevance, as natural variability in chemical cues from different individuals could have influenced the response. Similar findings were reported for *Epipedobates anthonyi* tadpoles, which showed no observable behavioral changes when exposed to injury-related chemical cues, both from individuals of the same species and from different species (Lipkowski et al. 2024). According to the authors, in certain ecological contexts, particularly in small, low-flow aquatic habitats such as temporary ponds, the transmission of chemical signals may be constrained by their low directionality and slow diffusion rate, reducing their effectiveness compared to other sensory modalities such as visual or tactile cues (Lipkowski et al. 2024). This is particularly relevant for the species evaluated in the present study, as they inhabit temporary ponds in Amazonia, where such environmental constraints are likely to influence sensory communication and predator-prey dynamics.

The effects of the stressors on cortisol differed between the two species. Only *S. ruber* exhibited marked elevations in cortisol in response to both temperature and methomyl exposure, as well as a non-additive increase under their interaction. Physiological challenges such as elevated temperature and pesticide exposure can activate the hypothalamic–pituitary–interrenal (HPI) axis in amphibians, triggering a hormonal cascade initiated by corticotropin-releasing hormone (CRH) release from the hypothalamus. CRH stimulates adrenocorticotropic hormone (ACTH) release from the pituitary, which in turn induces glucocorticoid secretion (e.g., cortisol, corticosterone) by interrenal glands (Santymire et al. 2018; Barough et al. 2025). These glucocorticoids play essential roles not only in acute stress responses but also act synergistically with thyroid hormone (TH) to regulate growth and metamorphosis in tadpoles. This interaction can accelerate metamorphosis in hostile environments as a strategy to escape unfavorable conditions. However, such acceleration often results in smaller post-metamorphic body size, potentially compromising performance (Denver 2009). During the larval stage, continuous feeding is critical to accumulate energy reserves for metamorphic climax, a phase marked by intense morphological and physiological changes. Stress-induced behavioral changes, such as reduced foraging, can hinder this energy acquisition (Orlofske and Hopkins 2009). Moreover, sustained cortisol elevation may deplete glycogen, protein and lipid stores, reducing the energy available for metamorphosis (Mommsen et al. 1999; Rocha et al. 2010). This aligns with the glucocorticoid-fitness hypothesis (CORT-fitness), which proposes that environmental stressors that promote prolonged increases in glucocorticoid concentrations may ultimately reduce organismal fitness, particularly in scenarios of high energetic demand. The hypothesis underscores the importance of considering not only the presence of an endocrine response, but also its duration and intensity when assessing the ecological and physiological impacts of stress in vertebrates (Bonier et al. 2009). Amphibian species differ in their primary circulating glucocorticoid; while corticosterone predominates in many, some tropical frogs primarily secrete cortisol (Denver 2009; Santymire et al. 2018). Thus, the absence of detectable cortisol variation observed in *O. taurinus* may not indicate the absence of a stress response but rather a physiological difference in the dominant hormone profile, which could possibly reflect a greater reliance on corticosterone. This contrasting pattern, where one species exhibited significant behavioral and biochemical alterations without corresponding changes in cortisol, while the other maintained physiological and behavioral stability yet showed marked cortisol elevation, emphasizes the complexity and species-specific nature of endocrine stress responses in amphibians.

### Study limitations

Although we adopted a sublethal concentration equivalent to 10% of the 96-h LC50, a conventional metric widely used in ecotoxicology to standardize sublethal assessments, this concentration may not reflect actual exposure levels in Amazonian aquatic environments. Existing monitoring data from other regions indicate that methomyl can occur in surface waters and agricultural wells, but reported concentrations are generally low and highly variable (Carbo et al. 2008; Van Scoy et al. 2013). No information is available for Amazonian water bodies, where pesticide monitoring is sporadic and does not capture short-term contamination pulses associated with rainfall or runoff. This lack of continuous monitoring complicates the interpretation of ecological relevance because occasional peak concentrations may remain undetected (Chow et al. 2020). Therefore, while the 10% LC50 approach provides a standardized sublethal reference, its correspondence with real exposure scenarios in Amazonian systems remains uncertain. The absence of corticosterone quantification is a limitation that could be addressed in subsequent studies to complement the interpretation of the endocrine effects of methomyl-induced stress. Regarding the alarm substance preparation, it is important to note that there is a wide variety of protocols, and this is an area that needs further standardization and understanding to ensure comparability across studies. Lastly, the linear mixed models showed instability, with random-effect (aquarium) variance estimated as zero in most analyses, and permutation-based inference was used for statistical validation. This acknowledgment is important for properly contextualizing the robustness of the results and preventing overly deterministic interpretations.

## Conclusion

This study suggests that *Osteocephalus taurinu*s and *Scinax ruber* tadpoles exhibit distinct behavioral and physiological responses to combined environmental stressors, including methomyl exposure and elevated temperature. Consistent with our first prediction, both stressors influenced neurophysiological and endocrine pathways, but in species-specific directions. *O. taurinus* exhibited temperature-dependent modulation of AChE activity, with reduced activity at 30 °C and a pronounced enzymatic increase under the combined exposure to methomyl and elevated temperature, whereas no detectable cortisol variation was observed. In contrast, *S. ruber* maintained stable AChE activity but showed significant cortisol elevation in response to both stressors, with a non-additive interaction effect. As anticipated in our second prediction, these physiological shifts were accompanied by behavioral alterations in *O. taurinus*, which displayed reduced locomotion and dark-substrate preference, patterns indicative of antipredator strategies, while S. ruber maintained behavioral stability, suggesting higher resilience. Contrary to our third prediction, neither species exhibited significant behavioral responses to alarm cues, indicating that short-term sublethal exposure may have masked chemosensory responses.

These interspecific contrasts likely reflect distinct ecological adaptations and glucocorticoid regulation, with *O. taurinus* potentially favoring corticosterone and *S. ruber* primarily cortisol. Importantly, our study provides the first evidence of how synergistic stressors affect Amazonian amphibians, a region critically underrepresented in ecotoxicological research. The Amazon harbors unparalleled biodiversity yet faces escalating threats from both climate change and contamination, underscoring the urgent need for region-specific studies that capture species-level vulnerabilities. Furthermore, our results reinforce the importance of multi-stressor approaches in ecological risk assessment, as interactions between contaminants and abiotic stressors can profoundly alter species’ responses beyond predictions based on isolated exposures.

Overall, these findings highlight that species-specific traits, particularly endocrine physiology and ecological history, must be considered when evaluating the effects of anthropogenic stressors on tropical amphibians, with direct implications for conservation and management in rapidly changing Neotropical ecosystems.

### Ethics statement

All the experiments were approved by the Ethics Committee on Animal Use of the National Institute for Amazonian Research (INPA) (process No. 83/2023). The animals were collected and transported under authorization from SISBIO-ICMBio (No. 80447-4).

## Data Availability

The datasets generated and/or analyzed during the current study are not publicly available but are available from the corresponding author upon reasonable request.
